# Mixed Reality as an Environment for Reaction Speed Assessment in Physically Active Young Adults: Measurement Reliability and Participant Experience Compared with Immersive Virtual Reality

**DOI:** 10.3390/s26175658

**Published:** 2026-09-06

**Authors:** Jacek Polechoński, Małgorzata Dębska-Janus, Karolina Kostorz, Michał Rozpara, Izabela Zając-Gawlak, Agnieszka Nawrocka, Jarosław Cholewa

**Affiliations:** Institute of Sport Sciences, Academy of Physical Education in Katowice, 40-065 Katowice, Poland; m.debska@awf.katowice.pl (M.D.-J.); k.kostorz@awf.katowice.pl (K.K.); m.rozpara@awf.katowice.pl (M.R.); i.zajac-gawlak@awf.katowice.pl (I.Z.-G.); a.nawrocka@awf.katowice.pl (A.N.); j.cholewa@awf.katowice.pl (J.C.)

**Keywords:** mixed reality, immersive virtual reality, reaction speed, reaction time, measurement reliability, video see-through, flow state, sense of presence

## Abstract

Mixed reality (MR) delivered through video see-through headsets could enable reaction-speed assessment while preserving awareness of the physical environment, but its measurement properties remain unclear. This study assessed the reliability of reaction-speed measurements in MR and compared performance and user experience with immersive virtual reality (VR). Forty university students (20 women; age 22.9 ± 2.3 years) completed three 1 min Reaction–Micro Wall trials in each environment in counterbalanced order using Meta Quest 3 and Rezzil Player. Mean reaction speed (RS) and the number of hits per minute (hits/min) were analyzed; enjoyment, flow, and presence were assessed with validated questionnaires. Single-trial intraclass correlation coefficients were good in both environments (0.797–0.884; all *p* < 0.001) and slightly higher in MR. RS (VR: 517.4 ± 44.6; MR: 521.9 ± 60.3 ms; *p* = 0.600), hits/min (*p* = 0.742), and presence (*p* = 0.577) did not differ significantly. VR elicited greater enjoyment (*p* = 0.011) and flow (*p* = 0.003). Cross-environment associations were moderate (RS r = 0.494), and Bland–Altman analysis showed negligible bias (−4.6 ms) but wide limits of agreement (−111.3 to +102.2 ms). MR enables reliable group-level reaction-speed assessment; no significant differences between the environments were detected, although equivalence was not formally tested, and longitudinal monitoring of individuals should be conducted consistently within one environment.

## 1. Introduction

Reaction speed is one of the fundamental coordination abilities and—alongside movement accuracy—serves as a measure of the quality of motor behavior. It is understood as the rate of the body’s response to a stimulus, and its overall value consists of two components: reaction time (RT) and movement time (MT). The former is the interval from the occurrence of an unexpected stimulus until the initiation of the appropriate response, whereas the latter is the time from the beginning to the completion of a planned movement [1,2]. Depending on the number of stimuli and required responses, a distinction is made between simple reaction time (SRT), in which one signal corresponds to one action, and choice reaction time (CRT), which requires differentiated responses to two or more stimuli [3]. An increase in the number of possible choices logarithmically increases decision-making time, as described by Hick’s law [4]. Response efficiency depends primarily on the speed of information processing in the central nervous system, with stimulus identification representing its longest phase; this process is modified by numerous factors, such as age, sex, fatigue, mental state, and individual predispositions [5].

Conventional tools for assessing reaction speed lie between two extremes. Laboratory tests, based on responding by pressing a key to a stimulus appearing on a monitor, provide high measurement precision, but involve only fine motor skills, are usually performed in a seated position, and differ from the movement patterns used in sports competitions. Their reliability may be moderate: a meta-analysis of computerized neurocognitive batteries showed that, for many reaction-time-based tasks, reliability coefficients do not exceed values regarded as satisfactory [6]. Field tests, such as the ruler drop method, are simple and inexpensive, but are burdened by low precision and dependence on the examiner, who initiates the stimulus themselves—after several repetitions, the participant begins to anticipate the moment at which the instrument will be dropped [7]. Therefore, the search for solutions combining measurement precision with global, sport-specific movement patterns has become a natural direction for the development of coordination ability assessment.

A partial response to these needs is provided by the so-called light sport training systems (LSTSs), including FitLight Trainer™ and BlazePod™, as well as stationary reaction boards such as Dynavision D2. The reliability of such tools generally falls within the range indicative of good reliability: intraclass correlation coefficients of 0.75–0.92 have been reported for the Dynavision D2 board [8], similar values for batteries of reaction and cognitive-load tests [9], and values ranging from 0.833 to 0.884 for reactive agility tests in young soccer players [10]. Similar results were obtained for the BlazePod system in a group of mixed martial arts athletes, in which the coefficients ranged from 0.785 to 0.833 and the results correlated significantly with classic computer-based tests [11]; similar values were also reported in other study groups [12,13]. However, such systems have important limitations. They require pods to be physically placed in the testing space, and participants—out of fear of contact with a hard component—may reflexively decelerate the movement in its final phase [14]. Moreover, the issue of ecological validity has been raised: response speed to a light stimulus poorly predicted reactive agility measured under game-like conditions, indicating that responding to a light signal and responding to an opponent’s movement are not equivalent [15].

Immersive virtual reality makes it possible to overcome some of these limitations. Sensors located in the headsets and controllers enable precise recording of movement parameters, virtual objects can be positioned at any point in space, the procedure is fully standardized and independent of the examiner, and the user remains isolated from interfering external stimuli. The potential of this technology in sport has been the subject of an increasing number of review papers [16,17,18], and experimental studies indicate that skills acquired in a virtual environment can be transferred to real-world conditions—this has been demonstrated, among other things, for reactive behaviors in karate kumite [19,20] and in perspective reviews addressing the learning of complex motor tasks [21]. The first validated diagnostic tools have also been developed. An original test of simple and complex reaction implemented in VR achieved reliability comparable to computer-based tests and correlated significantly with them [22], transferring the ruler-drop method to a digital environment made it possible to eliminate the influence of the examiner on the result [7], and another solution made it possible—unlike LSTSs—to separate reaction speed into RT and MT components for global movement patterns [14]. Similar conclusions emerge from the validation of an entire battery of motor tests in VR [23] and of a simple reaction test [24], whereas a study in which cognitive tasks were performed in a faithful digital replica of a real room found no differences compared with real-world conditions [25].

One limitation of most of the aforementioned studies is their reliance on prototype solutions unavailable to practitioners. This gap is filled by the validation of the commercial Rezzil Player application, operating in a virtual environment, in which the results of the “Reaction” module were compared with measurements performed using the SMARTfit device in a real environment; high reliability was obtained in both conditions and significant correlations were found between the methods, while a systematic prolongation of reaction time in VR of up to 101.6 ms was also noted [26]. The direction of this difference is surprisingly consistent in the literature [7,22,24], and hardware latency—estimated at 21–42 ms for headsets of this class [27]—only partially explains it, although even small controller delays have been shown to impair aiming accuracy in a virtual environment [28]. Additional mechanisms have therefore been proposed: altered perceptions of depth, distance, and spatial orientation in digital environments [29,30], altered gaze behavior [31], the novelty effect, and cognitive load resulting from the multisensory nature of VR. The problem, however, is that all these studies simultaneously compared two different tools and two different tasks, rather than the environments themselves. Determining whether the difference is attributable to environmental virtuality or to the measurement approach requires a study design in which the device, application, task, and outcome measures are held constant, while only the degree of virtuality is varied.

Such an opportunity is provided by mixed reality (MR). According to the classic concept of the virtuality continuum, intermediate environments combining real and computer-generated elements fall between the fully real and fully virtual poles of this continuum [32]. Contemporary consumer headsets, such as Meta Quest 3, implement MR using video see-through (passthrough) technology: cameras transmit a color image of the surroundings to the displays, within which virtual objects are spatially anchored. Thus, the same device and the same application can present the same task once in full immersion and once against the background of a real room. Applications of MR in motor assessment and therapy are developing dynamically—in a review of 105 studies, head-mounted displays were found to currently be the most common hardware solution, alongside a shortage of comparative studies [33]. A classic manual dexterity test has already been compared with its VR and MR counterparts in post-stroke patients [34], and in analytical tasks, VR was found to outperform augmented reality in terms of sense of presence, speed, and accuracy of performance [35]. However, with regard to reaction speed, there are no data on either whether MR affects its magnitude or whether a measurement performed in MR is reliable. This question has practical relevance: retaining a view of the real environment increases participant safety, makes it possible to conduct measurements in the presence of and in verbal contact with the coach, and allows digital tasks to be combined with actual sports equipment.

Motor assessment in digital environments is not limited to performance indicators alone. The level of activity enjoyment, intensity of the flow state, and sense of presence jointly determine participant engagement and, consequently, their readiness to perform a trial at maximum intensity and their willingness to repeat tests over a longer period. These aspects are assessed, respectively, using the PACES scale [36], the short version of the Flow State Scale [37], and the Presence Questionnaire [38,39]. Previous studies have shown that physical activity in an immersive environment is rated as attractive and enjoyable and sometimes surpasses conventional exercise in this respect [40,41]; under some conditions, it even provides greater enjoyment and a stronger flow state than analogous activity in the real world [42]. Training using commercial immersive applications, in turn, improves visuomotor coordination and reaction time [43], whereas forms combining physical exertion with cognitive tasks in VR promote the development of visuomotor abilities [44]. An open question, however, remains: how the mixed environment compares in this context. From a theoretical perspective, complete isolation from external stimuli should promote a higher sense of presence in VR, but high-quality video see-through and the spatial anchoring of virtual objects may eliminate this difference. User comfort is also important because simulator-sickness symptoms reduce both the sense of presence and cognitive–motor performance [45,46].

Potential factors differentiating the results require separate consideration. In large population samples, men have shorter simple and choice reaction times than women [5]; however, in homogeneous groups of physically active individuals, these differences may be small, especially when a task involves global movement patterns rather than finger motor skills alone. Similarly, reports concerning laterality are inconclusive: some authors report shorter reaction times for the dominant hand, others for the non-dominant hand, and these discrepancies are related to task complexity and cerebral hemispheric specialization [4,47]. In tests based on a 3 × 3 board, in which the participant independently decides which hand to use to reach for a given stimulus, an indicator of the lateral distribution of hits additionally emerges alongside reaction time, which has so far received virtually no analysis and which, in the present study, is therefore treated as an exploratory outcome.

In summary, the available data confirm the reliability and validity of reaction speed measurements in immersive VR, whereas mixed reality remains an unexplored environment in this respect. It is unknown whether a measurement performed under video see-through conditions has comparable repeatability, whether the degree of environmental virtuality itself—when the tool and task remain unchanged—modifies the test result, or how both digital environments are evaluated by participants themselves. Filling this gap has practical significance because it determines whether reaction speed assessment can be conducted without isolating the athlete from the real environment.

The main aim of the study is to assess the reliability of reaction speed measurements in young adults performed in mixed reality (MR) and to compare the results obtained with measurements conducted in an immersive virtual reality (VR) environment, using the Rezzil Player application and Meta Quest 3 headsets. In addition, the level of physical activity enjoyment, intensity of the flow state, and sense of presence were assessed in both digital environments. Because the evidence reviewed above, the test task, and the studied group all originate from the context of sport and physical activity, the scope of this work is deliberately limited to young, physically active adults, which is also reflected in the title of the paper.

Four research questions were formulated to address this aim. First, it was examined whether reliable reaction speed measurements can be performed using the Rezzil Player application in both MR and VR environments. Second, it was investigated whether the type of digital environment differentiates the reaction speed of the study participants. Third, it was verified whether sex constitutes a factor differentiating reaction speed in immersive environments. Finally, it was sought to determine which of the digital environments—virtual or mixed—provides, in the participants’ opinion, a higher level of activity enjoyment, a stronger flow experience, and a greater sense of presence.

## 2. Materials and Methods

### 2.1. Study Participants

The study was conducted at the Laboratory for Research on Health-Oriented Physical Activity of the Jerzy Kukuczka Academy of Physical Education in Katowice, operating within the PN-EN *ISO 9001:2015*; Quality management systems—Requirements. International Organization for Standardization: Geneva, Switzerland, 2015. The study group comprised 40 students of the university (age: 22.9 ± 2.3 years; range: 19–29 years; body mass: 76.2 ± 16.8 kg; body height: 178.1 ± 9.5 cm), including 20 men (age: 22.6 ± 2.6 years; body mass: 86.3 ± 16.0 kg; body height: 184.8 ± 6.6 cm) and 20 women (age: 23.1 ± 1.9 years; body mass: 66.1 ± 10.4 kg; body height: 171.4 ± 7.0 cm). Thirty-seven participants reported being right-handed and three left-handed.

The inclusion criteria were good general health; no contraindications to the use of immersive technologies (in particular, no history of motion sickness, epilepsy, or hypersensitivity to flashing light); no history of neurological disorders or injuries that could affect sensorimotor function (e.g., multiple sclerosis, traumatic brain injury, stroke, or peripheral neuropathy); no current functional limitations (e.g., injuries); refraining from intense physical exercise during the 12 h preceding the measurement; and no use of substances or medications that could affect rapid responding. Before the study began, each participant was informed of its aim and procedure, provided written consent to participate, and signed an information clause concerning data processing; participants were also informed of their right to withdraw at any stage of the study without giving a reason. The study was conducted in accordance with the principles of the Declaration of Helsinki, and its procedure was approved by the Bioethics Committee of the Academy of Physical Education in Katowice (protocol 9/2018 dated 19 April 2018, together with amendment KB/27/2022 dated 25 October 2022).

### 2.2. Apparatus and Reaction Speed Measurement Procedure

**Figure 1:**
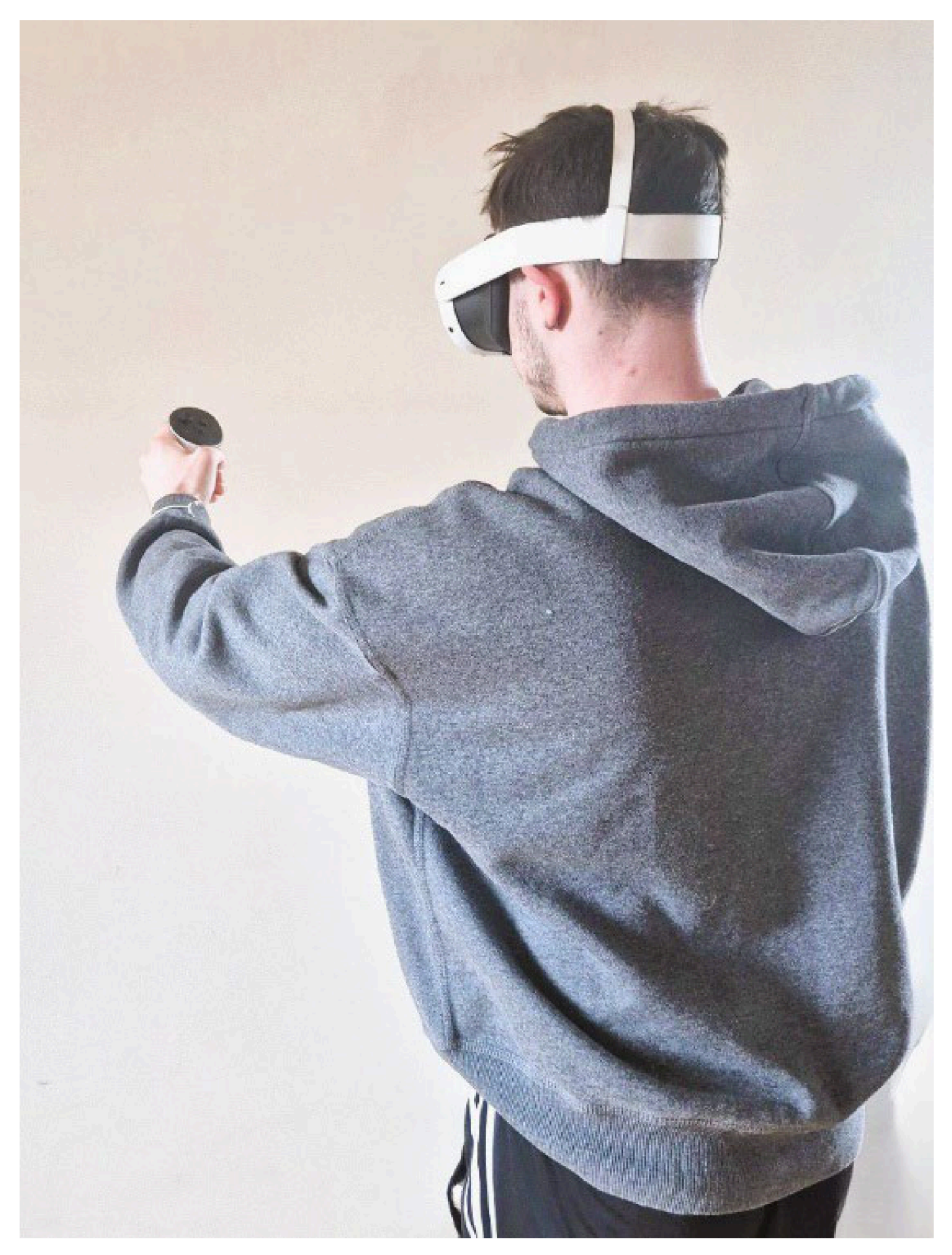
Study participant during a reaction speed test performed using the Rezzil Player application and Meta Quest 3 headsets.

All measurements were conducted under standardized laboratory conditions: with constant ambient lighting, without acoustic stimuli that could interfere with the course of the test, and at a temperature of approximately 22 °C. Standalone Meta Quest 3 headsets (Meta Platforms, Menlo Park, CA, USA), together with controllers, were used for image display, whereas the commercial Rezzil Player application (Rezzil, Manchester, UK), available from the Meta Store, was used to perform the test task, specifically the “Reaction” module and the “Micro Wall” option with a board arranged in a 3 × 3 grid. Figure 1 shows a study participant while performing the test.

The task consisted of touching, as quickly as possible, a target that lit up red and was positioned in one of the nine fields of the board, using controllers held in the right and left hands. After a hit, the target went out and another one lit up in a randomized manner. Participants performed the task in a standing position, at a distance from the board that allowed them to freely reach all nine targets with both hands; the choice of the hand used to reach for a given stimulus was left to their discretion. Each trial lasted 60 s, and participants performed three trials in each environment, separated by five-minute breaks. Before the actual measurements began, the digital environment was calibrated and one familiarization trial was conducted. The application recorded, with an accuracy of 0.001 s, the mean interval between the illumination of a target and contact with it, the total number of hits, and the number of hits performed with each hand. The means from the three trials were used for further analyses. The interval recorded by the application therefore comprises both the reaction time and the movement time of the upper limb; for this reason, and although the Rezzil Player interface labels this output “reaction time”, it is referred to throughout the present paper as mean reaction speed (RS), consistently with the definitions adopted in the Introduction and with the earlier validation of this module [26]. The 60 s trial duration, the number of trials, and the length of the breaks replicate the protocol used in that validation study, in which the results of the Rezzil Player were compared with the SMARTfit system serving as the reference method [26]; this duration provides a number of responses sufficient for a stable mean value while keeping the time of continuous headset use short enough to avoid visual or muscular fatigue.

**Figure 2:**
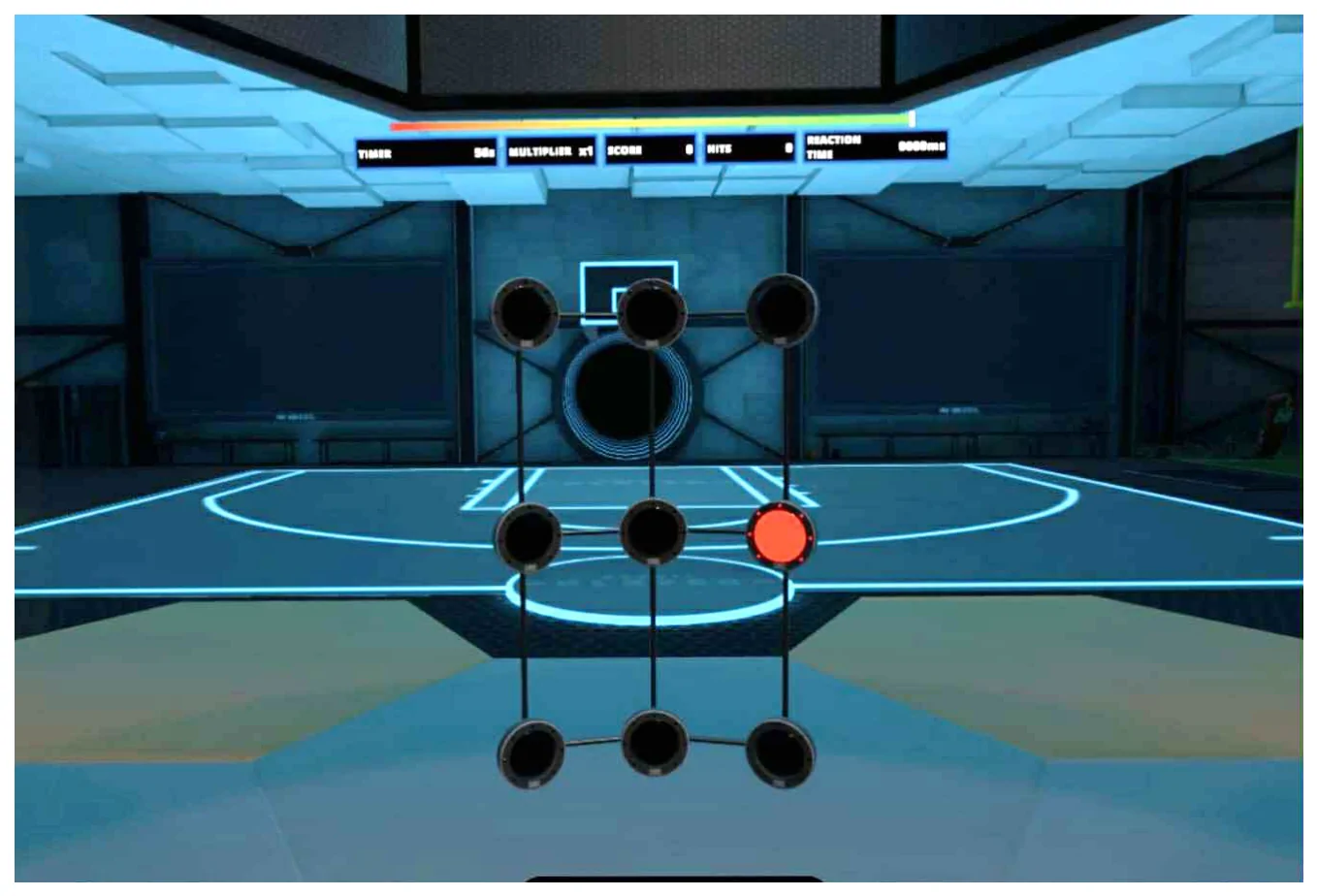
View seen by the user during a test performed in immersive virtual reality—a 3 × 3 board with one illuminated target.

**Figure 3:**
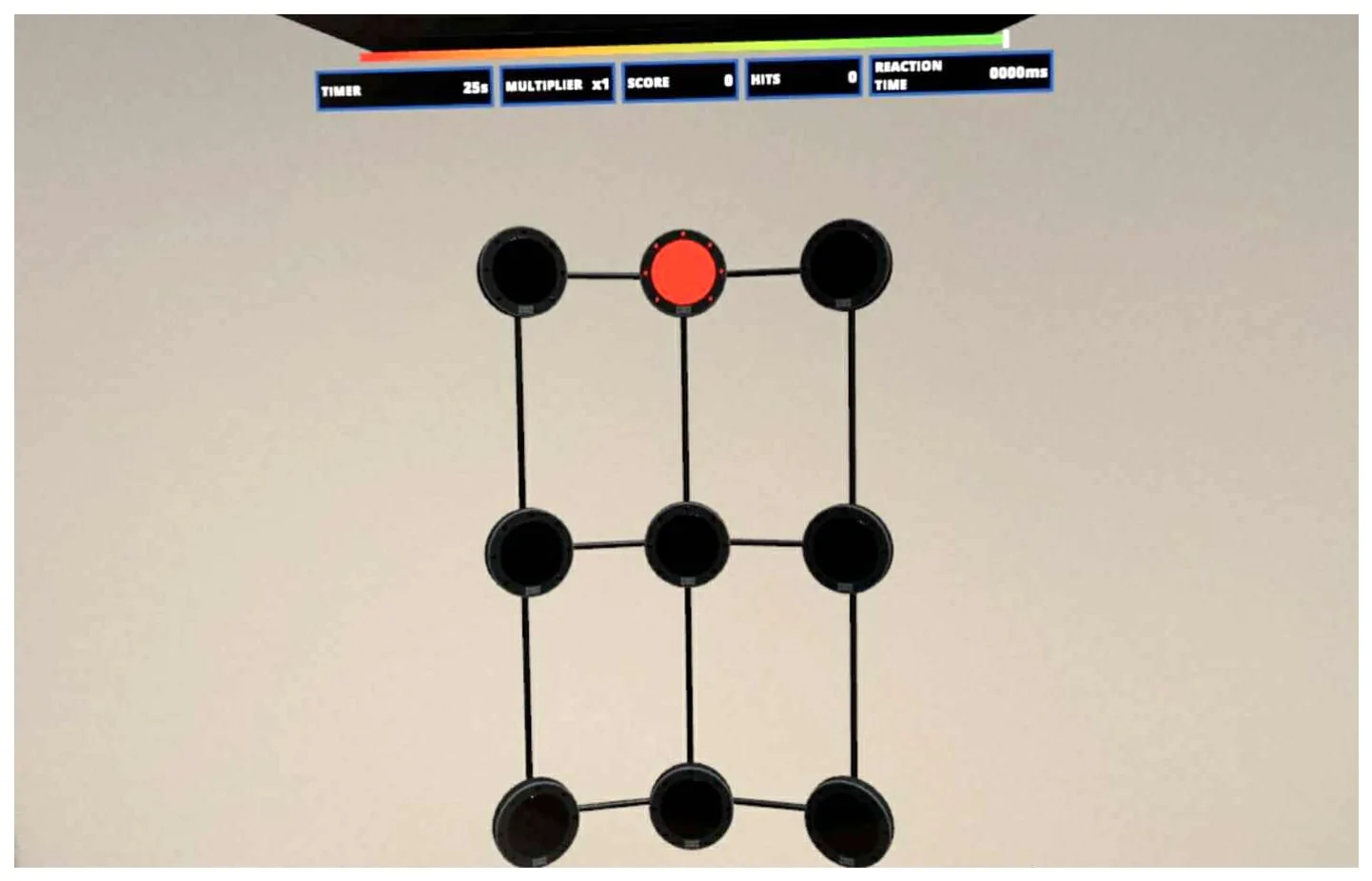
View seen by the user during a test performed in mixed reality—the same 3 × 3 board spatially anchored in the real interior of the laboratory.

Measurements were conducted in two digital environments. Under immersive virtual reality conditions, the board was presented in a fully computer-generated setting that isolated the user from visual stimuli in the surroundings (Figure 2). Under mixed reality conditions, the video see-through function (color passthrough) was activated, which allowed the virtual board to be spatially anchored in the real interior of the laboratory, remaining within the participant’s field of view (Figure 3). Apart from the degree of environmental virtuality, all elements of the procedure—the device, application version, task configuration, trial duration, and recorded indicators—were identical, which made it possible to isolate the effect of the environment from the effect of the measurement instrument. For the same reason, a condition based on optical see-through augmented reality was not included: it would have required a different headset and would thus have reintroduced the very hardware differences that the present design was intended to eliminate. Because both environments were generated by the same device and the same application, any additional latency of the video see-through pipeline would appear as a systematic difference between the environments and would be detected in the Bland–Altman analysis (Section 3.3).

To limit the effect of order, a counterbalanced design was applied: half of the participants (*n* = 20) began the study with VR and then proceeded to trials in MR, whereas the other half (*n* = 20) performed the trials in the reverse order. A 10 min break was maintained between the measurement blocks in both environments. Participants could discontinue a trial at any time if they experienced discomfort; after completion of testing in each environment, they were asked about symptoms of simulator sickness (dizziness, nausea, eye fatigue, and headache).

### 2.3. Assessment of Participants’ Experiences

After completing the trials in a given environment, participants completed three questionnaires referring exclusively to that environment. Enjoyment of the performed physical activity was assessed using the short form of the Physical Activity Enjoyment Scale (PACES-SF), consisting of 8 items rated on a scale from 1 to 7 [36]. The intensity of the flow state was measured using the Flow State Scale (FSS-2), comprising the nine dimensions of flow, with a five-point response scale ranging from 1 (“strongly disagree”) to 5 (“strongly agree”) [37]. Sense of presence was assessed using the 24-item version of the Presence Questionnaire (PQ), with a scale ranging from 1 to 7 and comprising items related to involvement, sensory fidelity, adaptation and immersion, and interface quality, including auditory and haptic experiences [38,39]. Reverse-worded items were reverse-coded before calculating the scores. Mean scores for each scale were used in the analyses.

### 2.4. Statistical Analysis

Forty individuals with complete results in both environments were included in the analysis (women: *n* = 20; men: *n* = 20). For each environment, the final result was the mean of three 1 min trials. Distributions were described using the arithmetic mean (M) and standard deviation (SD), whereas the normality of distributions and distributions of differences were verified using the Shapiro–Wilk test.

Before recruitment began, an a priori analysis of the required sample size was conducted in G*Power 3.1 [48] for the primary comparison of mean reaction speed obtained in the VR and MR environments, planned using a paired-samples Student’s *t* test. A two-sided significance level of α = 0.05, test power of 1 − β = 0.80, and a moderate effect size of *d*_z_ = 0.50 were adopted. Due to the lack of previous studies directly comparing both environments using an identical task, an effect of moderate practical significance was conservatively assumed. The calculations indicated a minimum required sample size of 34 participants. Taking into account the possible loss of up to 15% of data or participant withdrawal, the recruitment of at least 40 individuals was planned.

The reliability of repeated trials was assessed using the intraclass correlation coefficient ICC(3,1) in a two-way mixed-effects model, with participant as a random effect and trial position as a fixed effect, for a single measurement and using an absolute-agreement definition, in accordance with the procedures of Koo and Li [49]. For each coefficient, the 95% confidence interval, Fisher–Snedecor F statistic, and *p*-value were reported. ICC values were interpreted as follows: <0.50—poor reliability, 0.50–0.75—moderate reliability, 0.75–0.90—good reliability, and >0.90—excellent reliability.

Comparisons of results obtained in VR and MR were performed using a paired-samples Student’s *t* test. Effect size was expressed as Cohen’s *d* for paired samples (*d*_z_) or the rank-biserial correlation coefficient (r_rb_). The strength of the association between results from both environments was estimated using Pearson’s correlation coefficient (r), or Spearman’s correlation coefficient (r_S_) for variables with distributions deviating from normality, whereas absolute agreement was assessed using the Bland–Altman method, reporting systematic bias (the mean VR − MR difference) and 95% limits of agreement (LoA = bias ± 1.96 × SD of the differences) [50]. The association between two indicators of the same ability—mean reaction speed and the number of hits—within each environment was assessed using Spearman’s correlation. In addition, 95% confidence intervals were calculated for the mean VR−MR differences. Neither an equivalence nor a non-inferiority test was planned, and therefore the absence of statistically significant differences is not interpreted as evidence of equivalence between the environments. Changes across the three consecutive trials were additionally examined using one-way repeated-measures analysis of variance, with partial eta squared (ηp^2^) as the measure of effect size.

Comparisons between women and men within each environment were conducted using an independent-samples Student’s *t* test and, in the absence of normality, the Mann–Whitney U test, with appropriate effect size measures (Cohen’s *d*, r_rb_). The lateral distribution of hits, expressed as the percentage of hits performed with the left hand, was compared with 50% using a one-sample *t* test. All tests were two-sided, with a significance level of α = 0.05. Calculations were performed using IBM SPSS Statistics (IBM Corp., Armonk, NY, USA). This last analysis was exploratory in nature and was not covered by the primary research questions.

## 3. Results

### 3.1. Measurement Reliability

All participants had complete results in both environments; none discontinued the study or reported symptoms that might indicate simulator sickness. The reliability of a single trial was good for both analyzed parameters in each environment, and all coefficients were statistically significant (Table 1). In VR, ICC values were 0.826 for mean reaction speed and 0.797 for the number of hits, whereas in MR they were slightly higher, reaching 0.884 and 0.869, respectively. The lower limits of the confidence intervals exceeded 0.68 in all cases, meaning that even under the most conservative estimate, measurement reliability remained at least moderate. Performance did not change systematically across the three consecutive trials in either environment (one-way repeated-measures ANOVA: F(2,78) = 0.77–1.84; *p* = 0.165–0.465; ηp^2^ = 0.019–0.045), which indicates that a practice effect did not contribute substantially to the reported reliability coefficients.

### 3.2. Comparison of Results Obtained in VR and MR

The distributions of VR−MR differences for the five primary variables did not deviate significantly from normality (Shapiro–Wilk: *p* = 0.054–0.616); therefore, a paired-samples Student’s *t* test was applied. No significant differences were found in mean reaction speed, the number of hits, or sense of presence. However, significantly higher scores were obtained in VR on the PACES and FSS scales than in MR; the effect sizes for both effects were small to moderate (Table 2). For the two primary outcomes, the 95% confidence intervals for the mean VR−MR difference were −22.0 to +12.9 ms for reaction speed and −3.2 to +4.5 hits/min. These intervals should be read as an absence of a detectable difference rather than as evidence of equivalence between the environments.

### 3.3. Agreement of Measurements Performed in Both Environments

**Figure 4:**
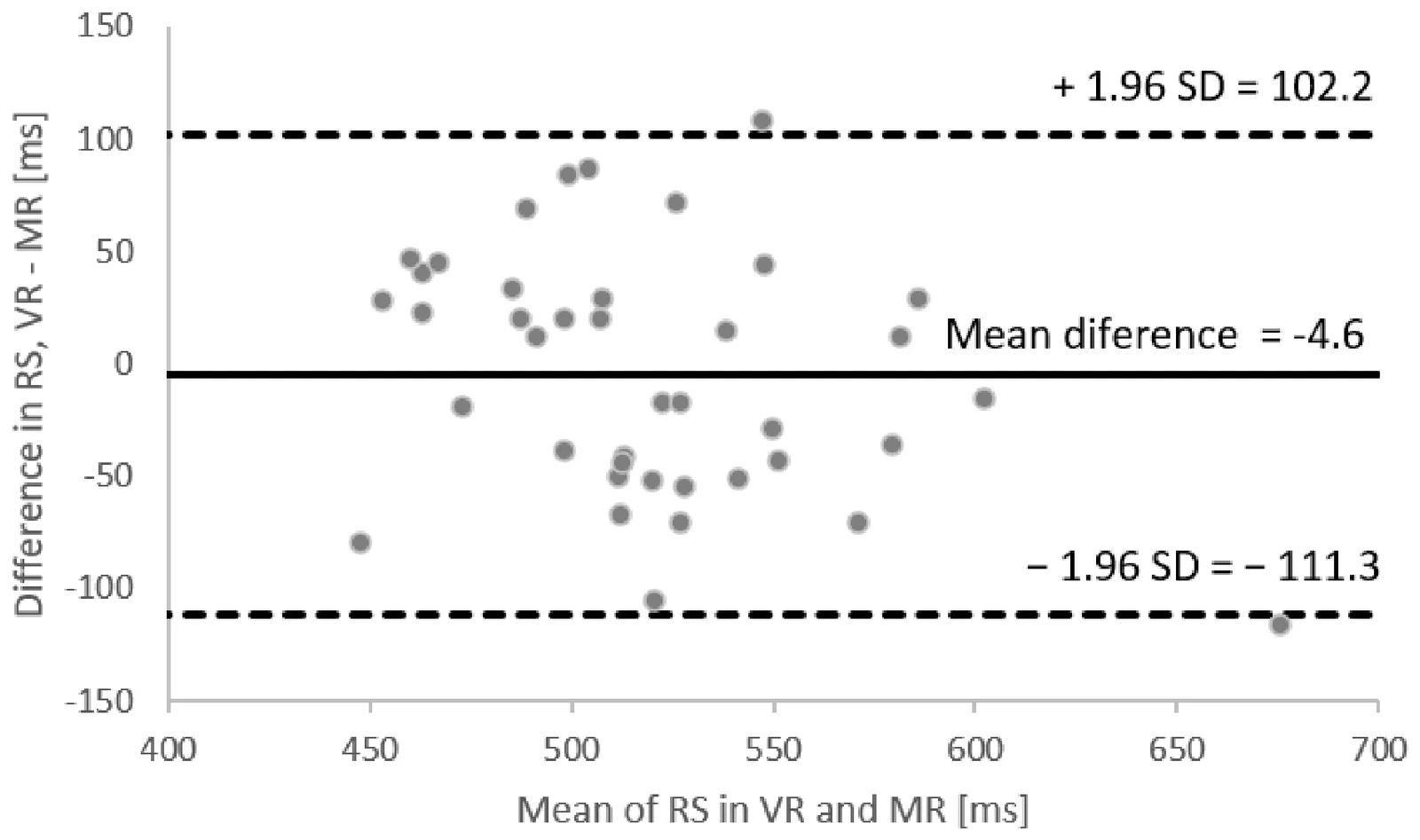
Bland–Altman plot for mean reaction speed (RS) obtained in immersive virtual reality (VR) and in mixed reality (MR) (*N* = 40). Each point represents one participant. The solid line indicates the mean difference (bias, VR − MR = −4.6 ms) and the dashed lines the 95% limits of agreement (LoA, −111.3 to +102.2 ms).

**Figure 5:**
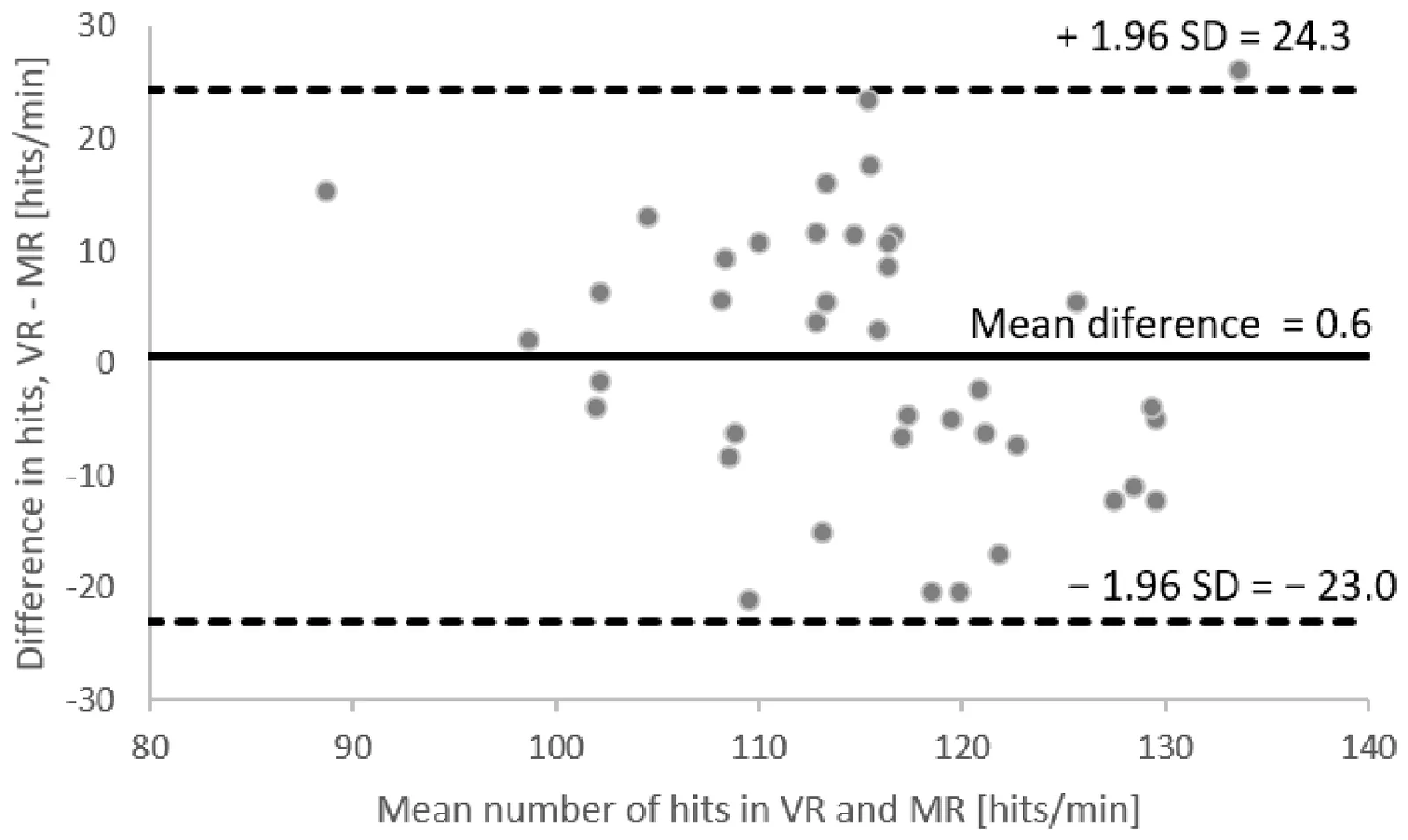
Bland–Altman plot for the number of hits obtained in immersive virtual reality (VR) and in mixed reality (MR) (*N* = 40). Each point represents one participant. The solid line indicates the mean difference (bias, VR − MR = +0.6 hits/min) and the dashed lines the 95% limits of agreement (LoA, −23.0 to +24.3 hits/min).

The absence of differences at the group mean level does not imply the interchangeability of individual results. The correlation between results obtained in both environments was moderate and statistically significant—for mean reaction speed, r = 0.494 (*p* = 0.001), and for the number of hits, r = 0.431 (*p* = 0.006). Bland–Altman analysis demonstrated very small systematic bias (VR − MR): −4.6 ms for reaction speed and +0.6 hits/min for the number of hits, with wide 95% limits of agreement ranging, respectively, from −111.3 to +102.2 ms and from −23.0 to +24.3 hits/min (Table 3; Figure 4 and Figure 5). This means that, at the population level, both environments yield virtually identical results, whereas an individual participant’s result may differ markedly between them. Both analyses are presented graphically in Figure 4 and Figure 5. In each case, the differences are scattered symmetrically around a bias line running close to zero and no funnel-shaped pattern is visible; the slight negative slope of the differences across the range of measured values (b = −0.40; *p* = 0.036 for reaction speed) corresponds to the greater dispersion of the results recorded in MR. The limits of agreement correspond to ±20.5% of the mean value for both indicators.

### 3.4. Association Between Reaction Indicators

The two primary indicators reported by the application proved to be almost completely redundant. The correlation between mean reaction speed and the number of hits was very strong and negative both in immersive VR (r_S_ = −0.982; *p* < 0.001) and in MR (r_S_ = −0.996; *p* < 0.001). This is a consequence of the fixed-duration task design, in which the number of hits is, in practice, a conversion of mean reaction speed. From the perspective of diagnostic practice, both parameters can be used interchangeably, and reporting both simultaneously does not provide additional information about the ability being assessed.

### 3.5. Lateral Distribution of Hits

In an exploratory analysis, study participants, who independently decided which hand to use to reach for a given stimulus, performed 41.5 ± 14.6% of hits with the left hand in VR and 43.1 ± 14.2% in MR. In both environments, this percentage was significantly lower than 50% (VR: t(39) = −3.70; *p* < 0.001; MR: t(39) = −3.09; *p* = 0.004), indicating a clear predominance of the right hand in performing the task—consistent with the fact that 37 out of 40 participants reported right-handedness. The difference between environments was not significant (*p* = 0.900), and this indicator showed the highest cross-environment consistency among the analyzed variables (r = 0.699; *p* < 0.001), suggesting that it reflects a stable, individual movement preference of the participant rather than a property of the environment.

### 3.6. Comparison of Women and Men

In both environments, no significant differences were found between women and men in mean reaction speed or the number of hits (Table 4). Due to the deviation from normality of the reaction speed distribution in the group of women, the Mann–Whitney U test was used for this variable, whereas an independent-samples Student’s *t* test was used for the number of hits. Men achieved nominally better results (by 17.5 ms in VR and 13.2 ms in MR), but the magnitudes of these effects remained small to moderate. Sex also did not differentiate any of the questionnaire variables: for all six comparisons (PACES, FSS, and PQ in VR and in MR), *p* > 0.30, with an effect size not exceeding 0.30.

### 3.7. Summary of Results

The “Reaction—Micro Wall” test demonstrated good reliability of single trials in both VR and MR. No evidence was found that the type of digital environment differentiated the primary indicators of reaction speed; no sex-related differences were observed either. However, agreement between results at the individual level was only moderate. Participants experienced greater enjoyment of the activity and higher flow in VR than in MR, with a comparable sense of presence in both environments.

## 4. Discussion

The present study—to our knowledge, one of the first to address reaction speed assessment in MR—indicates that this environment allows this ability to be assessed at least as reliably as fully immersive VR. The obtained ICCs (0.797–0.884) correspond to good reliability according to the classification of Koo and Li (2016) [49] and fall within the range reported for the same application module in a virtual environment and for an analogous test performed using the SMARTfit device under real-world conditions [26]. They are also close to the values obtained for other diagnostic tools based on global movement patterns, such as the BlazePod system [11], proprietary reaction tests in VR [22], and solutions allowing reaction speed to be separated into its components [14]. It is worth emphasizing that these values are no lower, and often higher, than those reported for stationary devices and computerized test batteries: for the Dynavision D2 board, repeatability indices ranged from 0.75 to 0.92 [8], similar values were reported for novel reaction time and cognitive-load protocols [9], and a reaction test battery used in young athletes demonstrated reliability ranging from moderate to good [51]. By comparison, a meta-analysis of computerized neurocognitive tools indicated that a substantial proportion of reaction-time-based tasks did not reach the threshold regarded as satisfactory [6]. The slightly higher reliability in MR than in VR may result from the presence of a real background, which provides stable spatial references and facilitates calibration of the distance from the board between successive trials; however, this observation requires confirmation because the ICC differences were small and their confidence intervals overlapped.

The most important result of the study should be considered the absence of significant differences in reaction speed between the two versions of the digital environment. This result should be contrasted with consistent reports of prolonged reaction time in VR compared with the real environment or conventional computer tests—from a dozen or so to more than twenty milliseconds for simple reaction, through more than one hundred milliseconds for complex reaction [22], to 101.6 ms in a study using the same Rezzil Player application module [26]; a similar direction of the association has also been reported for other tools [7,24]. An interpretation based solely on technological latency does not fully explain these differences, because motion-to-photon latency for headsets of this class is 21–42 ms [27], although even small controller delays measurably impair the accuracy of aiming actions in a virtual environment [28]. In the present study, the device, application, motor task, and outcome measures were identical, and only the degree of environmental virtuality differed—and under these conditions, the differences proved negligible. This suggests that the previously described discrepancies may result, at least in part, from differences in measurement tools and task design rather than from the virtuality of the environment alone. It should be emphasized, however, that the present comparison covered two digital environments and did not include a real-world condition; a direct verification of this interpretation would require a design in which the same task and the same tool are also applied in a real environment. This observation is consistent with the results of studies in which reaction-time-based cognitive tasks performed in a faithful digital replica of a real room did not differ from those performed under real-world conditions [25], while also explaining why comparisons involving different devices lead to opposite conclusions. This has direct practical significance: results obtained in VR and MR using the same tool can be compared and combined at the group level, for example, when establishing norms or conducting an aggregate assessment of intervention effects.

However, the individual level requires a more cautious interpretation. The moderate correlation between environments and the relatively wide limits of agreement show that individual results are not fully interchangeable. The coexistence of virtually zero systematic bias with a wide dispersion of differences is typical of a situation in which within-person variability and measurement error outweigh the effect of the factor under study [50]. This phenomenon is not specific to digital environments—similar observations were made when augmented reality, immersive VR, and a conventional clinical test were compared in the assessment of manual dexterity, where, despite the good psychometric properties of the individual test versions, their mutual interchangeability proved limited [34]. Therefore, in diagnostic practice, the monitoring of an individual athlete’s progress should be conducted consistently in one environment, after prior adaptation to the tool, and comparisons of results obtained in different headset operating modes require caution. The width of the limits of agreement should therefore be related to the intended purpose of the measurement. At the group level, the estimate of the difference was precise (95% CI −22.0 to +12.9 ms), which is sufficient for comparing conditions or establishing normative values. At the individual level, the limits of agreement (±20.5% of the mean value) exceed the changes usually expected after a training intervention, and for this reason we do not regard them as acceptable for using the two environments interchangeably when monitoring a single participant; the same applies to the number of hits.

The strong negative association between mean reaction speed and the number of hits confirms previous observations concerning the same application module [26]. From a psychometric perspective, such a high correlation between two indicators of the same construct constitutes evidence of convergent validity [52], yet it also indicates complete informational redundancy. This results from the design of a fixed-duration task, in which the number of hits is almost an exact conversion of the reciprocal of mean reaction speed. In scientific reporting, mean reaction speed, expressed in milliseconds and therefore directly comparable with the results of conventional tests, should be recommended; the number of hits remains a more intuitive indicator in communication with the athlete.

No sex-related differences were found, although men obtain shorter simple and choice reaction times than women in large population samples [5]. This finding can be explained by several factors. The study group comprised students of a sports-oriented university and was therefore homogeneous in terms of age and physical activity level. Moreover, the applied task involved global movement patterns—reaching with the upper limbs to targets positioned in space—rather than finger motor skills alone, so anthropometric differences may have partly compensated for potential differences in the speed of stimulus processing. Men nevertheless achieved nominally better results, and the effect size for reaction time in VR was small to moderate, which, given subgroup sizes of 20 participants each, means that the absence of differences should be regarded as a preliminary conclusion requiring confirmation in a larger sample.

An exploratory addition to the analysis is the lateral distribution of hits. Participants who independently decided which limb to use performed only 41–43% of hits with the left hand, significantly less than would be expected under an equal distribution. The predominance of the dominant hand corresponds to observations concerning reaction time asymmetry between the two hands [4,47], but in this case it pertains not so much to reaction speed as to the strategy used to solve the task. The high consistency of this indicator across environments suggests that it reflects a stable, individual movement preference. This may make it a useful tool for monitoring the symmetry of actions in coordination training, especially in sports requiring bilateral proficiency; however, its diagnostic value requires separate verification.

The assessment of participants’ experiences revealed a clear, although moderate, advantage of the fully immersive environment. Higher scores on the PACES and FSS scales in VR are consistent with reports according to which physical activity in an immersive environment is rated as attractive and enjoyable, sometimes more highly than conventional exercise with a comparable load [40,41], and under some conditions provides greater enjoyment and a stronger flow state than analogous activity in the real world [42]. This has significance beyond participant comfort alone, because positive experiences promote engagement and maintenance of activity, and forms combining physical exertion with cognitive tasks in an immersive environment lead to measurable improvements in visuomotor abilities [43,44].

However, the comparable level of sense of presence in both environments is surprising, because complete isolation from external stimuli should theoretically promote a stronger sense of “being there.” This result differs from those obtained in analytical tasks, in which the sense of presence was significantly higher in VR than in augmented reality [35]; however, that study was based on headsets with optical see-through and a narrow field of view, whereas the high-resolution video see-through applied here, together with the spatial anchoring of virtual objects, may have generated a comparable sense of immersion. The limitations of the tool should also not be overlooked: the PQ was developed for fully virtual environments, and some of its items—concerning the realism of scenery or interface quality—refer ambiguously to mixed conditions [38]. The nature of the task, which was brief and strongly attention-demanding, may have additionally limited the possibility of exploring the surroundings and becoming aware of their properties. It is also worth noting that spatial perception in a digital environment is not identical to perception in the real world: differences in spatial orientation between real and virtual conditions [29], different patterns of gaze behavior during motor skill learning [31], and the impact of the presentation medium on upper-limb movement performance [30] have been demonstrated. The fact that the reaction test result remained unchanged despite these differences strengthens the conclusion that the applied task is robust to changes in the degree of environmental virtuality.

From a practical perspective, the study results lead to two complementary recommendations. Because MR provides measurement reliability no lower than VR and does not modify the test result, it may be preferred when participant safety, the possibility of conducting measurements in direct contact with a coach, and combining digital tasks with actual sports equipment are important—thus, under typical diagnostic conditions in a sports club or hall. Immersive virtual reality, in turn, appears more advantageous where maximum participant engagement and positive experiences are crucial, primarily in training applications whose effectiveness in shaping reactive behaviors and transferability to real-world conditions have already been documented [18,19,21]. The development of video see-through technology makes this distinction increasingly practical because both options are now available within a single, relatively inexpensive device. It should be remembered that—regardless of the environment—tests based on responding to a light stimulus measure basic perceptual–motor abilities rather than the ability to respond to an opponent’s actions; therefore, they should complement, rather than replace, sport-specific tests [15].

## 5. Study Limitations

The presented results should be interpreted with consideration of several circumstances arising from the adopted study design. The study group comprised young, physically active students of a sports university, which ensured high homogeneity and limited the influence of confounding variables; at the same time, however, it narrows the extent to which the obtained values can be generalized to other populations. Their verification among children, older adults, or high-level athletes constitutes a natural direction for further work. The study was conducted using one headset model and one application, which was necessary to maintain constant comparison conditions, but this means that extending the conclusions to other devices requires separate confirmation. System latency in the two operating modes was also not measured directly; although both modes were implemented using the same device and application, and the observed differences proved negligible, supplementing the protocol with the measurement of motion-to-photon latency would make it possible to rule out this factor unequivocally. The design did not include either a real-world condition or an optical see-through augmented reality condition, and therefore the conclusions apply only to the comparison of two digital environments. In addition, the sample size was determined so as to detect a moderate effect, and no equivalence or non-inferiority test was planned; the results should therefore be read as an absence of detectable differences and not as evidence of equivalence.

Symptoms of simulator sickness were monitored on an ongoing basis and none of the participants reported them; however, the use of a validated questionnaire tool would provide more precise data in this regard. Similarly, the Presence Questionnaire, originally developed for fully virtual environments, remains the most well-documented tool of its kind, but its results with regard to mixed reality require a more cautious interpretation, and the development of tools adapted to mixed environments would facilitate future comparisons. Reliability was estimated on the basis of three consecutive trials within one session, which corresponds to diagnostic practice in club settings and is consistent with procedures adopted in analogous validation studies; extending this assessment to an analysis of between-day repeatability would be a valuable development. Finally, participants responded by hitting targets using controllers held in their hands, which is a standard solution in tests of this type and ensures high recording accuracy, although the development of hand-tracking technology—already used in the assessment of manual dexterity [34]—opens the possibility of an even more faithful representation of natural movement.

## 6. Conclusions

The relatively high values of intraclass correlation coefficients obtained for variables characterizing reaction speed during tests performed in MR indicate that this environment enables reliable measurements of this motor ability to be conducted, and the values obtained in MR were no lower than those observed in the fully immersive environment. No significant differences in participants’ reaction speed were found between environments, and the size of the environmental effect was negligible. Within the precision of the present study, no differences in reaction speed related to the type of digital environment were therefore detected, although the equivalence of the two environments was not formally tested. Given that all the remaining elements of the procedure were held constant, this result suggests that the differences between digital and real environments described in the literature may to a large extent result from differences in tools and test tasks; verifying this suggestion requires a design that also includes a real-world condition.

However, the comparability of the two environments at the group level does not fully translate into the interchangeability of individual results: the correlation between environments was moderate, and the limits of agreement were relatively wide. Therefore, changes in reaction speed in an individual participant should be monitored consistently in one environment. The collected data also did not show significant differences between women and men in reaction tests performed in both VR and MR, suggesting that, in a homogeneous group of young physically active individuals, sex does not constitute an important factor differentiating reaction speed in digital environments; however, given the moderate effect size and limited subgroup sample sizes, this conclusion requires confirmation in a larger sample.

Physical activity associated with performing reaction speed assessment tests in VR provides participants with greater enjoyment and a higher level of flow than the same test tasks in MR. This suggests that the immersive environment may create more favorable conditions for engagement and motivation, whereas MR—by providing equally reliable measurement while retaining contact with the surroundings—remains at least an equally useful solution for diagnostic purposes. The possible influence of these experiences on the effectiveness of training and on the development of motor abilities was not examined in the present study and requires separate verification. The comparable level of sense of presence in both digital environments may be surprising, as it seemed that immersive VR, owing to the greater isolation of users from external stimuli, should have been rated higher in this respect; explaining this observation requires the use of tools adapted to the specific characteristics of mixed environments.

## Figures and Tables

**Table 1:** Reliability of three repeated trials of the “Reaction—Micro Wall” test in both digital environments (*N* = 40).

Environment	Parameter	ICC (95% CI)	F	*p*
VR	Mean reaction speed (ms)	0.826 (0.728–0.897)	15.15	<0.001
VR	Number of hits (hits/min)	0.797 (0.687–0.878)	12.90	<0.001
MR	Mean reaction speed (ms)	0.884 (0.815–0.933)	24.41	<0.001
MR	Number of hits (hits/min)	0.869 (0.791–0.923)	20.99	<0.001

ICC(3,1)—two-way mixed-effects model for a single measurement; CI—95% confidence interval. For all analyses, F—Fisher–Snedecor test: df1 = 39, df2 = 78; *p*—probability value for F test.

**Table 2:** Comparison of results obtained in VR and MR (*N* = 40).

Parameter	VR: M (SD)	MR: M (SD)	t(39)	*p*	*d*_z_
Mean reaction speed (ms)	517.4 (44.6)	521.9 (60.3)	−0.53	0.600	−0.08
Number of hits (hits/min)	115.6 (10.0)	114.9 (12.3)	0.33	0.742	0.05
PACES (points)	5.97 (0.55)	5.74 (0.81)	2.68	0.011	0.42
FSS (points)	4.18 (0.46)	4.05 (0.47)	3.22	0.003	0.51
PQ (points)	5.29 (0.68)	5.26 (0.62)	0.56	0.577	0.09

M—mean; SD—standard deviation; t—paired-samples *t* test; *p*—probability value for *t* test; *d*_z_—Cohen’s *d* for paired samples. A positive *d*_z_ value indicates a higher score in VR than in MR.

**Table 3:** Association and agreement of results obtained in VR and MR (*N* = 40).

Parameter	r (Pearson)	*p*	Bias (VR − MR)	95% Limits of Agreement
Mean reaction speed (ms)	0.494	0.001	−4.6	−111.3 to +102.2
Number of hits (hits/min)	0.431	0.006	+0.6	−23.0 to +24.3

r—Pearson’s correlation coefficient; *p*—probability value for r; Bias—mean difference in results obtained in VR and MR; limits of agreement were calculated as bias ± 1.96 × SD of the differences.

**Table 4:** Comparison of reaction test results in women and men in VR and MR.

Parameter	Women: M (SD)	Men: M (SD)	Test	*p*	Effect Size
VR—mean reaction speed (ms)	526.1 (45.1)	508.6 (43.4)	U = 236.0	0.337	r_rb_ = 0.18
MR—mean reaction speed (ms)	528.5 (65.3)	515.3 (55.7)	U = 221.5	0.570	r_rb_ = 0.11
VR—number of hits (hits/min)	113.8 (9.1)	117.3 (10.8)	t(38) = −1.10	0.278	*d* = −0.35
MR—number of hits (hits/min)	113.4 (12.4)	116.4 (12.4)	t(38) = −0.77	0.449	*d* = −0.24

M—mean; SD—standard deviation; U—Mann–Whitney test; t—independent-samples *t* test; *p*—probability value for U/*t* test; r_rb_—rank-biserial correlation coefficient; *d*—Cohen’s *d* for independent samples. A positive r_rb_ or d value indicates a higher score for women.

## Data Availability

The raw data supporting the conclusions of this article will be made available by the authors on request.

## Abbreviations

The following abbreviations are used in this manuscript:

CI	Confidence interval
CRT	Choice reaction time
*d*	Cohen’s *d* coefficient
*d*_z_	Cohen’s *d* for paired samples
FSS	Flow State Scale
HMD	Head-mounted display
ICC	Intraclass correlation coefficient
LoA	Limits of agreement
LSTS	Light Sport Training Systems
M	Arithmetic mean
MR	Mixed reality
MT	Movement time
PACES	Physical Activity Enjoyment Scale
PQ	Presence Questionnaire
r	Pearson correlation coefficient
r_S_	Spearman correlation coefficient
r_rb_	Rank-biserial correlation coefficient
RS	Reaction speed
RT	Reaction time
SD	Standard deviation
SRT	Simple reaction time
VR	Immersive virtual reality
